# Use of different transmission metrics to describe malaria epidemiology in the highlands of western Kenya

**DOI:** 10.1186/s12936-015-0944-4

**Published:** 2015-10-26

**Authors:** Jennifer C. Stevenson, Gillian H. Stresman, Amrish Baidjoe, Albert Okoth, Robin Oriango, Chrispin Owaga, Elizabeth Marube, Teun Bousema, Jonathan Cox, Chris Drakeley

**Affiliations:** Department of Disease Control, Faculty of Infectious and Tropical Diseases, London School of Hygiene and Tropical Medicine, London, UK; Johns Hopkins Bloomberg School of Public Health, 615 North Wolfe St, Baltimore, MD 21205 USA; Department of Immunology and Infection, Faculty of Infectious and Tropical Diseases, London School of Hygiene and Tropical Medicine, London, UK; Radboud University Nijmegen Medical Centre, Nijmegen, The Netherlands; Kenya Medical Research Institute, Centre for Global Health Research, Kisumu, Kenya

**Keywords:** Malaria epidemiology, Highlands, Kenya, RDT, PCR, Serology

## Abstract

**Background:**

Monitoring and evaluation of malaria programmes may require a combination of approaches to detect any effects of control. This is particularly true at lower transmission levels where detecting both infection and exposure to infection will provide additional evidence of any change. This paper describes use of three transmission metrics to explore the malaria epidemiology in the highlands of western Kenya.

**Methods:**

A malariometric survey was conducted in June 2009 in two highland districts, Kisii and Rachuonyo South, Nyanza Province, Kenya using a cluster design. Enumeration areas were used to sample 46 clusters from which 12 compounds were randomly sampled. Individuals provided a finger-blood sample to assess malaria infection (rapid diagnostic test, PCR) and exposure (anti-*Plasmodium falciparum* MSP-1 antibodies) and a questionnaire was administered to record household factors and assess use of vector control interventions.

**Results:**

Malaria prevalence infection rates were 3.0 % (95 % CI 2.2–4.2 %) by rapid diagnostic test (RDT) and 8.5 % (95 % CI 7.0–10.4 %) by PCR and these ranged from 0–13.1 to 0–14.8 % between clusters for RDT and PCR, respectively. Seroprevalence was 36.8 % (95 % CI 33.9–39.8) ranging from 18.6 to 65.8 %. Both RDT and PCR prevalences were highest in children aged 5–10 years but the proportion of infections that were sub-patent was highest in those between 15 and 20 years of age (78.1 %, 95 % CI 63.0–93.3 %) and those greater than 20 years (73.3 %, 95 % CI 64.5–81.9 %). Those reporting both indoor residual spraying (IRS) in their home and use of bed nets had lower exposure to malaria compared to those who reported using IRS or bed nets alone.

**Conclusions:**

In this highland site in western Kenya malaria transmission was low, but highly heterogeneous. To accurately characterize the true extent of malaria transmission, more sensitive and complementary metrics such as PCR or serology are required in addition to the standard microscopy and/or RDTs that are routinely used. This is likely to be the case in other low endemicity settings.

**Electronic supplementary material:**

The online version of this article (doi:10.1186/s12936-015-0944-4) contains supplementary material, which is available to authorized users.

## Background

In recent years, much attention has been given to the debate as to whether malaria elimination is a viable and realistic option for malaria-endemic countries [1–3], whether the tools currently available are able to achieve elimination, and if elimination is a sensible strategy for control programmes [1]. What is clear is that metrics suited to lower transmission levels will be needed to allow accurate evaluation of control and elimination programmes [4]. The performance of different metrics needs to be characterized in a variety of medium and low transmission settings to ensure that the heterogeneity of malaria burden can be accurately described, targeted and monitored across operationally appropriate geographical scales.

Microscopy has been the historic mainstay of the estimation of infection prevalence. More recently infection prevalence has been assessed by rapid diagnostic tests (RDT). Both approaches have a similar sensitivity with limits of detection in the order of 5–50 parasites/μl [4]. Whilst the operational utility of these measures, particularly RDT, cannot be discounted, sufficient data have accumulated to suggest that they significantly underestimate the level of infection [5–7]. Molecular methods based on PCR of DNA detect 0.5–5 parasites/μl and reports suggest on average twofold more infections than that detected by microscopy [6, 8]. This difference is more pronounced in older individuals and in lower transmission settings and appears to be related in part to immunity and parasite density [5, 9]. The relevance of these low-density, sub-patent infections is that they can still infect mosquitoes. Furthermore, despite the typically lower density parasitaemia, the chronic and widespread nature of these infections suggests longer periods of infectivity, which therefore act as an important source of transmission especially in areas of high vector competence [5, 10].

In low transmission settings, the burden of malaria shifts with infections distributed across age groups. There have been few studies comparing the utility of different diagnostics in these settings [6–8, 11, 12]. There is increasing evidence that serological measures of exposure (i.e., the detection of antibody responses specific for malaria parasites) can provide an additional, more sensitive measure of transmission in these low-endemic areas. Antibody responses measured as simple prevalence in Somaliland showed that almost one-fifth of people had serological evidence of exposure despite the absence of parasites by microscopy and that exposure as determined by antibody levels was associated with travel to nearby Ethiopia [13]. At larger scales, the seroconversion rate (SCR) calculated from age and antibody prevalence has been shown to correlate with other measures of transmission intensity and therefore may be more useful in low-endemic settings [4, 14].

To further understand malaria dynamics in a low transmission setting, a cross-sectional survey was carried out in a highland fringe area of western Kenya (altitude 1400–1600 m). Whilst several studies have reported malaria transmission from either lowland or highland areas of Nyanza Province [15–20], few have been carried out between these two extremes. The aims of the surveys were to (1) characterize the epidemiology of *Plasmodium falciparum* in this setting; (2) to assess the utility of different diagnostic tools, including RDTs, PCR, and the presence of antibodies to the parasite antigen MSP1-19; and, (3) to identify factors associated with malaria infections/exposures based on the three malaria metrics used.

## Methods

### Study site and survey procedure

Malaria surveys were conducted in the neighbouring highland districts of Kisii Central, Kisii South and Rachuonyo South, Nyanza Province, western Kenya as part of the Malaria Transmission Consortium project [21]. The total population of the study districts at the time of the 2009 national census was 863,000. The area is predominantly rural with subsistence agriculture being the main occupation. People of the Kisii and Luo ethnic group predominantly occupy Kisii Central and Kisii South (henceforth referred to as Kisii) and Rachuonyo, respectively. Malaria transmission is seasonal with two peaks following the bimodal rainfall pattern, the heaviest rainfall typically occurring between March and June, with a smaller peak in October/November each year. The main malaria vectors are *Anopheles funestus* and *Anopheles arabiensis*, and *P. falciparum* is the predominant malaria parasite.

Sample size was calculated to adequately define the prevalence of *P. falciparum* infection among different age groups with 95 % confidence, with 80 % power, and assuming a design effect of 2. Based on historical prevalence data for the field area and its vicinity, the prevalence of infection was assumed to be 15 % among children under 5 years of age (the smallest population age group being considered in this study). Assuming an average household size of six persons and a 15 % non-response rate, a total sample of 1273 people per district was required to in order to estimate age-specific prevalence to a precision of ±3 %.

**Fig. 1:**
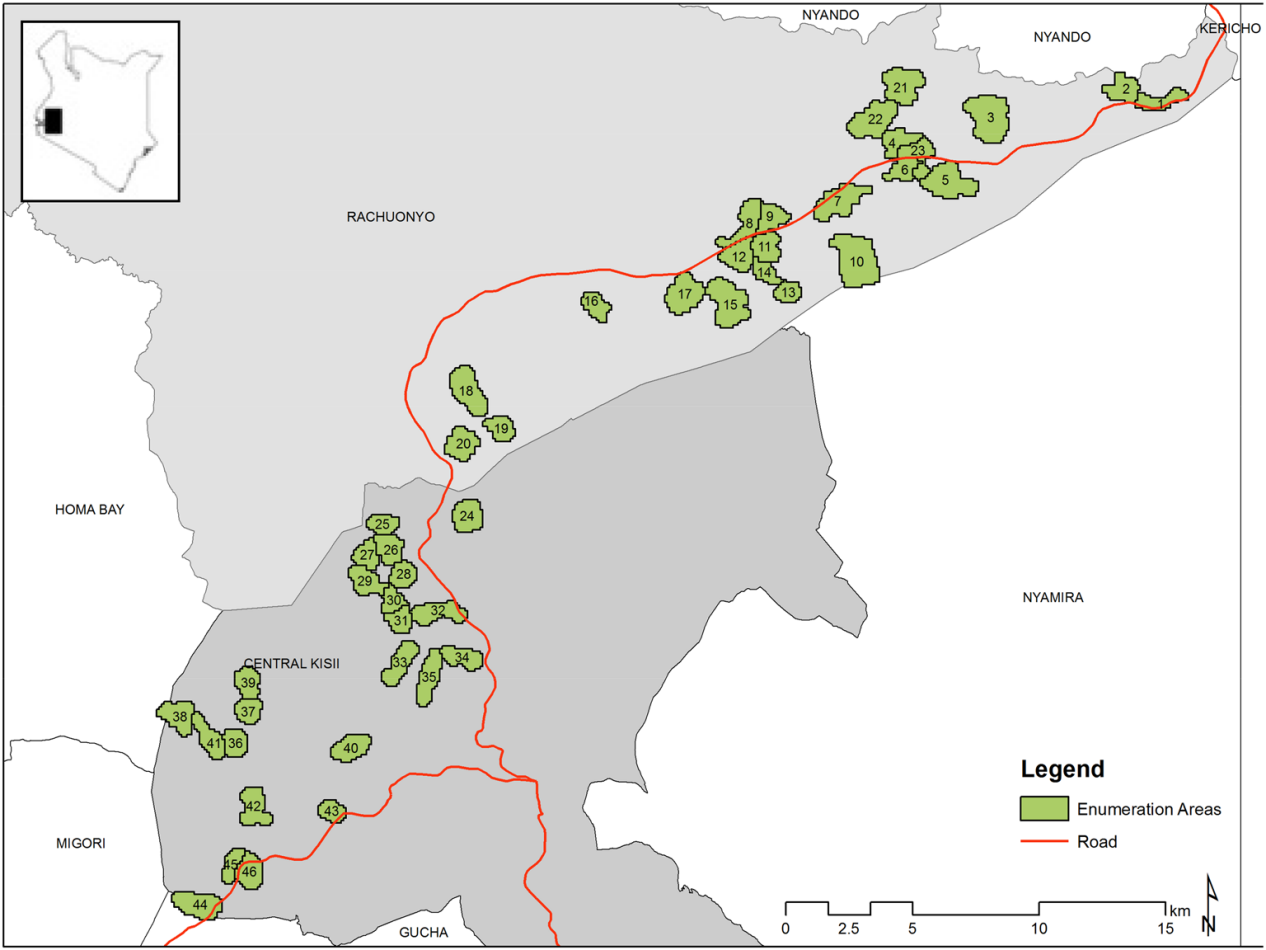
Map highlighting the location of the randomly selected enumeration areas included in the survey

Survey compounds (which represent a family homestead of one or more houses) were selected using a two-stage sampling design. At the first stage, 46 enumeration areas (EAs, the primary sampling unit) were selected randomly from a sampling frame restricted to EAs with a mean altitude of 1400–1600 m (Fig. 1). EAs were defined by the Kenya National Bureau of Statistics during the 2009 national census to demarcate areas with approximately 500 residents. Urban EAs and EAs with boundaries contiguous with the main Rachuonyo-Kisii boundary were excluded.

All compounds in selected EAs were geolocated and enumerated. Second stage sampling involved randomly selecting 12 compounds. All consenting occupants of the compound over the age of 6 months were eligible for inclusion in the study.

The survey was conducted in July 2009, at the end of the long rainy season. At each of the households, informed written consent was sought. The head of household was interviewed to assess household wealth indices and structure and individuals were questioned about malaria control behaviours. A finger-prick blood sample was taken from all consenting household members above the age of 6 months to measure parasite prevalence using PfHRP2 RDT (Paracheck Pf^®^, Orchid Biomedical Systems, Goa, India) and haemoglobin levels (HemoCue^®^, Angelholm, Sweden). Blood was also spotted onto filter paper (3MM Whatman^®^, Maidstone, UK) for subsequent molecular and serological analysis. Individuals with a positive RDT result were treated for malaria at the home according to national guidelines with artemether-lumefantrine (Coartem^®^, Novartis) or referred to a health facility if suspected to be pregnant or allergic to Coartem^®^. Participants found to be anaemic were provided haematinics according to national guidelines at the time of the survey.

### Ethics, consent and permissions

This study was approved by the ethical committees of the London School of Hygiene and Tropical Medicine and the Kenya Medical Research Institute (#SSC 1802). Approval was also provided by the Division of Malaria Control, Ministry of Public Health and Sanitation. Prior to the surveys, meetings were held with all district administrative representatives, chiefs and district health teams to inform them of the study and acquire local approval. Community meetings were held at each of the EAs to explain the methods of study to potential participants. Household heads were approached and the study explained to members of the home. Individual informed consent was sought from all residents of the compound above the age of 6 months by signature or thumbprint accompanied by the signature of an independent witness. Consent for children under the age of 18 was provided by a parent/guardian and children between 14 and 17 years also provided written assent by signature or thumbprint accompanied by the signature of an independent witness. As defined in the Kenya national guidelines, participants below 18 years of age who were pregnant, married, or a parent were considered ‘mature minors’ and consented for themselves [22].

### Laboratory procedures: ELISA

Antibody responses to *P. falciparum* were assessed by ELISA as previously described [23]. Briefly, recombinant protein *P. falciparum* MSP-1_19_ was coated on plates at a concentration of 0.5 µg/mL in coating buffer and incubated at 4 °C overnight. After blocking with 1 % (w/v) skimmed milk solution, the plates were washed, and samples were added in duplicate at a final dilution of 1:1000, with a pool of hyper immune serum titrated on each plate. Plates were incubated overnight at 4 °C and 50 µl of HRP-conjugated rabbit anti Human IgG (DAKO, #P0214) were added into each well and incubated. After a further series of washes, substrate solution (OPD, Sigma #P8287, in PBS) was added and the reaction was allowed to develop for 15–20 min before addition of stopping solution (2 M H2SO4). Raw optical density (OD) measurements were averaged and normalized against the positive control samples on each plate. The cut-off value of these assays to define seropositivity was determined using a mixture model, which considers those individuals with OD values greater than the cut-off determined by the model as being seropositive. Age adjusted SCR were calculated by fitting a simple reversible catalytic model using the seropositivity data to determine the rate at which individuals convert from seronegative to seropositive by year of age. The seroreversion rate (SRR) was fixed at a reversion rate of 4.057 for both population and cluster specific calculations. The SRR used was obtained according to estimates from previous studies, and was fixed to ensure robust estimates of SCR, particularly with the smaller sample sizes for the cluster-level estimates [14, 23, 24].

### Laboratory procedures: PCR

The presence of a current *P. falciparum* infection was assessed using nested PCR (nPCR). Blood-spotted filter papers were punched with a sterile hole punch and DNA was extracted using a Chelex-saponin method as described previously [25]. In brief, filter papers were incubated in a 0.5 % saponin-PBS solution overnight. Filter paper spots were washed once in a 1× PBS solution and DNA was eluted by boiling samples in a 6 % Chelex DNA/RNAase free solution. Extracted DNA was then added to two separate nPCR reactions targeting the ribosomal 18S [26] and a modified version targeting mitochondrial Cytochrome B region [27]. Gel electrophoresis was performed and samples were visualized on an ethidium bromide stained 2 % agarose gel. Samples were considered to be positive if they were positive in either assay.

### Statistical analysis

All statistical analysis was conducted in STATA (v 12.0, StataCorps, Texas, USA) using the survey (svy) command to account for the two-stage sampling design (EA and compound) and stratifying by district. Prevalence and means and the corresponding exact 95 % confidence interval (CI) were calculated assuming a binomial and normal distribution, respectively. Tests of significance were assessed using the Spearman’s and Chi square test as appropriate. Socio-economic status (SES) was generated using a principal component analysis (PCA) using household level wealth indicators collected as part of the questionnaire [28]. Indicators included education level attained by the head of household, house construction including wall, floor and roof type, as well as ownership of items such as animals, televisions, mobile phones, vehicles, and bicycles. To assess the association between the three outcome measures in this study, RDT, PCR and seroprevalence, logistic regression was conducting using the svy command as described above. Univariate analysis was conducted to determine the association with all potential risk factors. Next, a multivariable analysis was conducted in a backwards step-wise fashion retaining all variables significant at the 0.05 level. Variables included in the analysis were anaemia status (haemoglobin <11 g/dl), recent travel, the type of eaves present in the house, as well as the use of mosquito control measures [insecticide-treated nets (ITNs), indoor residual spraying (IRS)]. Finally, elevation was included as an ordinal variable with cut-offs defined using quartiles. The optimum model was determined using a likelihood ratio test comparing the saturated to the reduced model.

## Results

In total, 3566 people were sampled from 46 EAs with a mean of 78 (range 70–96) people sampled per EA (Table 1). Forty per cent (95 % CI 38.3–41.8, range 27.5–54.4) of the population sampled were ≥20 years of age, whilst a low proportion of samples came from those aged 15–20 years (7.4 %, 95 % CI 6.5–8.5 %, range 0–27.1 %). 56.3 % (95 % CI 54.7–57.9, range 47.3–68.0) were female. Use of bed nets was the most common form of malaria control with 60.3 % (95 % CI 58.7–61.9, range 12.3–91.7) of the population reporting having slept under a net the previous night. 24.0 % (95 % CI 19.6–29.1, range 0–70.4) of the population reported that they did not use bed nets nor had their house been sprayed in the past 12 months.

**Table 1:** Demographic data of all surveyed households

N = 3566	Value	95 % CI	Cluster range
Mean sampled per cluster	78	–	70–96
Median age (years)	13	–	9–22
Population sampled per age group (%)			
<5	22.5	21.2–23.9	12.5–31.2
5–9.9	17.4	16.1–18.7	7.8–27.6
10–14.9	12.6	11.2–14.2	4.0–27.1
15–19.9	7.4	6.5–8.5	0–27.1
≥20	40.0	38.3–41.8	27.5–54.4
Gender (% female)	56.3	54.7–57.9	47.3–68.0
Travelled outside EA in 3 months (%)	5.0	3.3–7.4	0–30.7
House has open eaves (%)	81.7	80.5–83.0	0–100
Socio-economic status (%)			
1 (low)	23.3	19.4–27.7	0–57.1
2	19.2	16.0–22.9	0–56.9
3	24.4	20.5–28.7	1.0–61.1
4	13.7	11.3–16.6	0–40.7
5 (high)	19.4	15.1–24.6	0–64.6
Mosquito control (%)			
Net only	29.7	24.9–35.0	0–76.9
IRS only	9.6	7.4–12.3	0–38.5
Both	30.6	25.1–36.7	0–85.5
None	24.0	19.6–29.1	0–70.4

Data includes the 95 % CI and range of values per cluster

Malaria infection by RDT was low overall with a mean prevalence of 3.0 % (95 % CI 2.2–4.2 %) but this ranged by EA from 0 to 13.1 % (Table 2). The malaria prevalence by PCR was over twice that of RDT with 8.5 % (95 % CI 7.0–10.4 %) being positive and 64.4 % (95 % CI 59.4–69.4 %) of PCR infections were sub-patent (i.e., PCR positive but RDT negative). The parasite prevalence by PCR was also highly heterogeneous with clusters ranging from 0 to 14.8 %. Exposure as assessed by antibody prevalence to malaria MSP antigen was 36.8 % (95 % CI 33.9–39.8 %) and varied by EA from 18.6 to 65.8 % (Table 2). Also, seropositivity in children <5 years old varied from 0 to 44 % across EAs suggesting heterogeneity in recent exposure across the study area. Mean SCR was 0.038 (95 % CI 0.035–0.040). There was a significant association between the rank of RDT and PCR prevalence at the EA level (Spearman’s p <0.001), but no association between SCR or seroprevalence and RDT (p = 0.185; p = 0.223) and PCR (p = 0.096; p = 0.114), respectively.

**Table 2:** Overall estimates of current malaria infection and exposure, as well as haemoglobin levels

	Mean	95 % CI	Cluster range
Proportion RDT positive	3.0	2.2–4.2	0–13.1
Proportion PCR positive	8.5	7.0–10.4	0–14.8
Proportion MSP-1_19_ antibody positive	36.8	33.9–39.8	18.6–65.8
MSP-1 SCR	0.038	0.035–0.040	0.013–0.128
Mean Hb level (g/dl)	12.8	12.7–12.9	11.8–13.8
Proportion anaemic (<11 g/dl)	25.7	23.1–28.5	9.4–48.0

Data given as means, corresponding 95 % CI and the range of values by cluster

Children aged 5–10 years had the highest malaria prevalence by both RDT (6.1 %, 95 % CI 4.2–8.0 %) and PCR (13.1 %, 95 % CI 10.4–15.7 %) (Table 3). However, older age groups were found to have the highest proportion of sub-patent infections; 78.1 % of PCR-confirmed infections in 15–20 year olds and 73.3 % of infections in those >20 years were sub-patent compared to 51.3 % in children <5 years (chi p = 0.015). Older children also reported the lowest bed net use; 55.6 % (95 % CI 51.0–60.2 %) and 56.5 % (95 % CI 50.6–62.5 %) in the 10–15 and 15–20 years age groups, respectively (Table 3).

**Table 3:** Malaria outcomes and factors commonly associated with malaria stratified by age group

	≤5 years		5.1–10 years		10.1–15 years		15.1–20 years		>20 years	
	%	95 % CI	%	95 % CI	%	95 % CI	%	95 % CI	%	95 % CI
Malaria outcomes										
RDT Pos	4.1	2.7–5.4	6.1	4.2–8.0	4.0	2.2–5.8	2.6	1.0–4.5	1.0	0.4–1.4
PCR Pos	7.8	5.9–9.7	13.1	10.4–15.7	10.4	7.5–13.2	10.6	6.9–14.4	6.0	4.8–7.2
Sub-patent	51.3	39.7–63.0	61.5	51.3–71.7	61.8	48.6–75.1	78.1	63.0–93.3	73.3	64.7–81.9
Malaria factors										
Bed net use	60.7	57.3–64.1	61.1	57.3–65.0	55.6	51.0–60.2	56.5	50.6–62.5	61.9	59.4–64.4
Travel	2.5	1.4–3.6	1.7	0.6–2.7	1.4	0.3–2.5	3.9	1.5–6.3	9.0	7.5–10.5
Anaemic	41.6	38.2–45.0	22.9	19.6–26.3	13.9	10.7–17.1	16.8	12.3–21.4	23.3	21.1–25.5

Data includes the corresponding 95 % CI

**Fig. 2:**
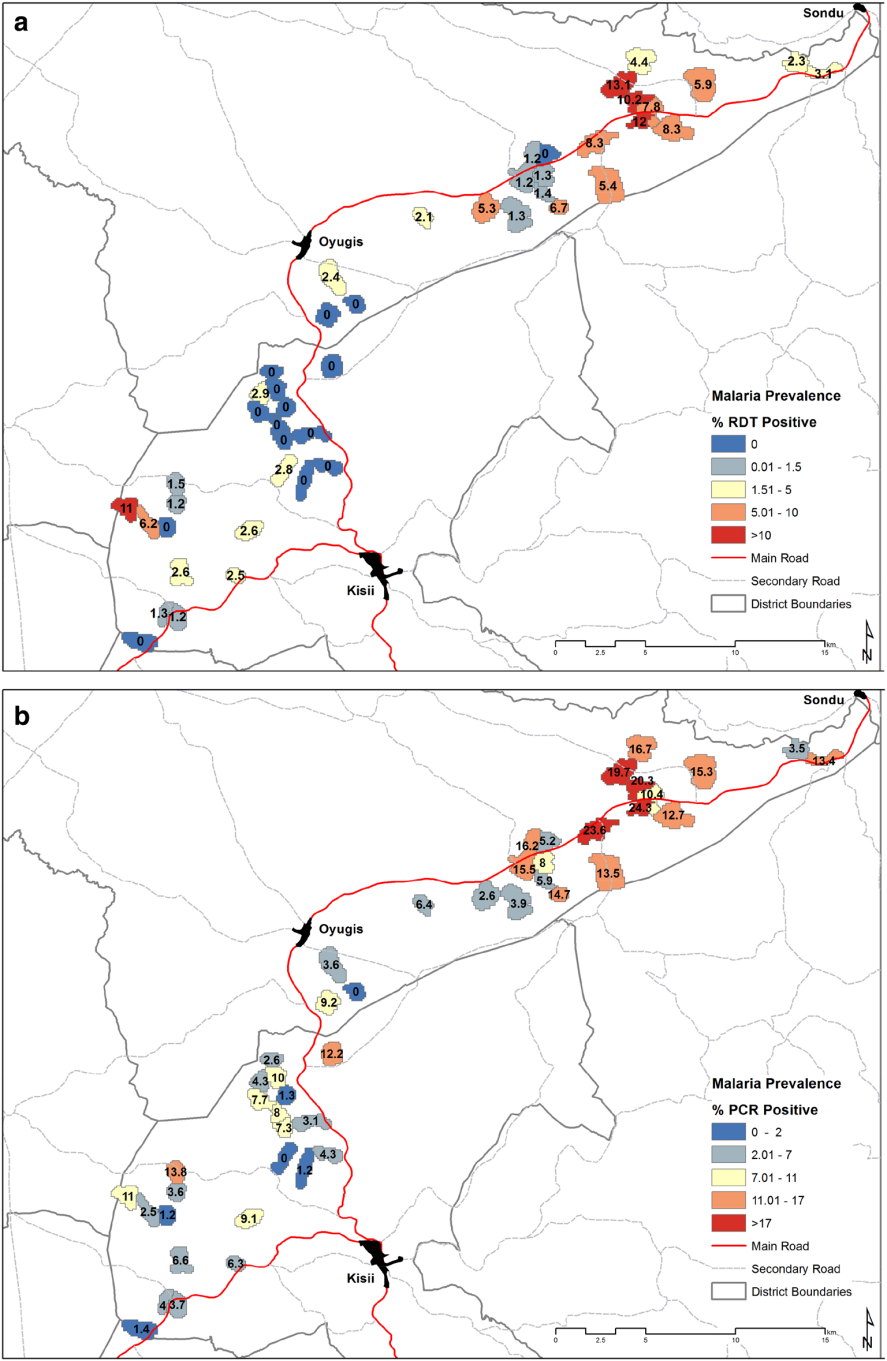
Map of EAs by **a** RDT prevalence, **b** PCR prevalence

**Fig. 3:**
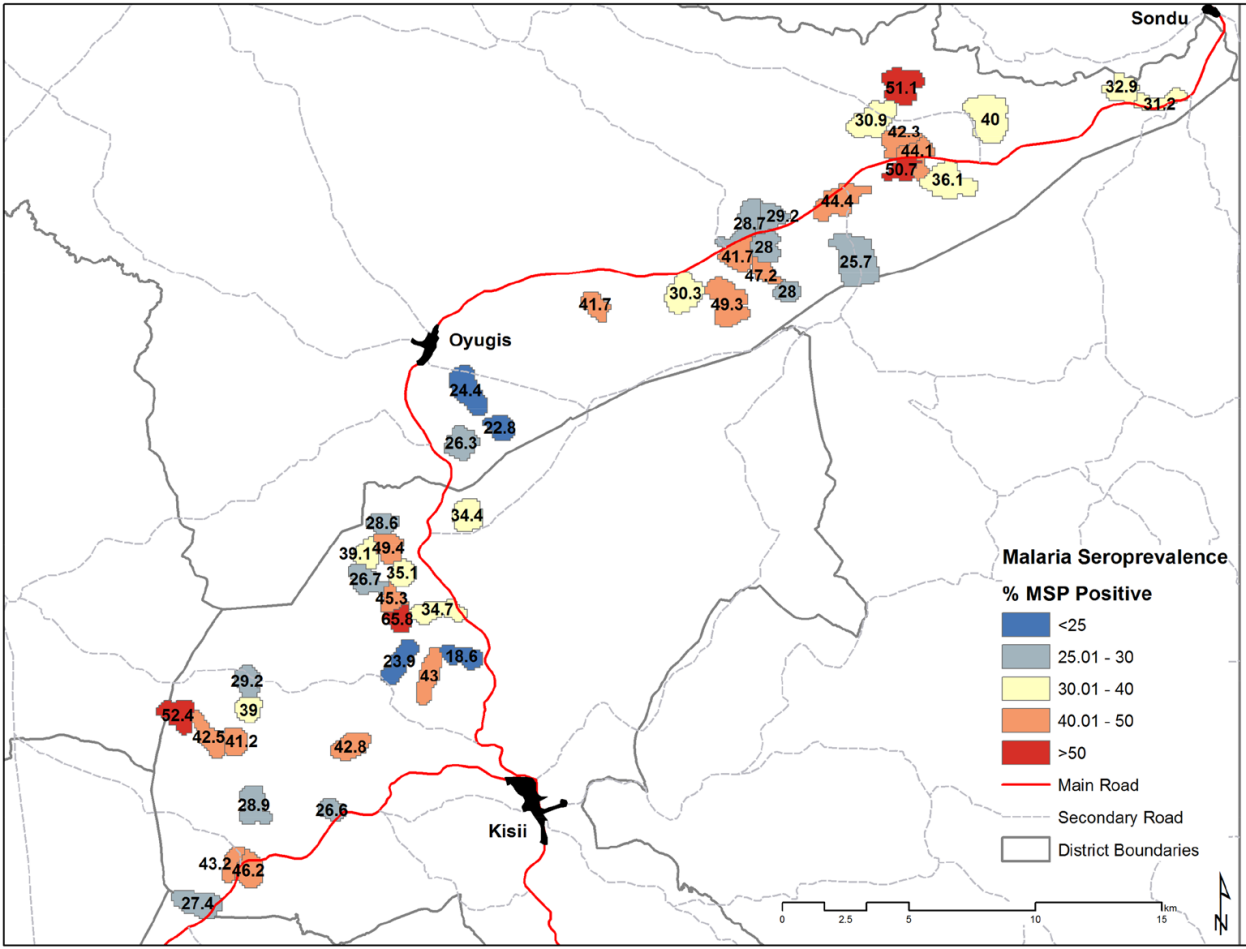
Map of EAs by seroprevalence

Of the 46 EAs included in the survey, 15 had no RDT-positive infections detected (Fig. 2a). In these clusters with no evidence of infection by RDT, the mean PCR prevalence was 5.0 % and this ranged between 0 and 12.2 % (Fig. 2b). Similarly, despite no RDT-positive individuals present, 32.5 % of individuals were positive for MSP antibodies and this ranged from 18.6 to 65.8 % (Fig. 3). In the 15 RDT-negative clusters, when seroprevalence estimates were restricted to those under 5 years of age, a proxy for recent transmission, 14.3 % of children were seropositive and this ranged from 0 to 43.7 %.

**Fig. 4:**
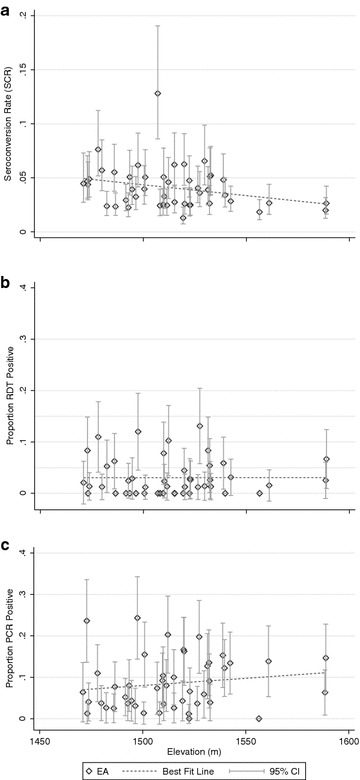
Scatter plots of malaria estimates per cluster by elevation, **a** seroconversion rate, **b** RDT prevalence, **c** PCR prevalence. Plots include 95 % CI

**Fig. 5:**
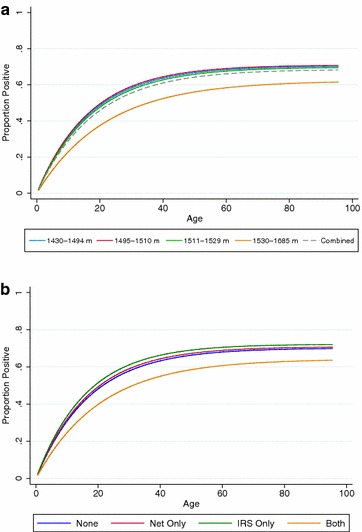
**a** Seroconversion curves per altitude band, **b** seroconversion curves per self-reported mosquito control

Variation according to EA altitude was observed for serological and parasitological outcomes. There was a large range in SCR across EAs (range: 0.013–0.128) and showed a non-significant negative trend with SCR decreasing by 0.0002 (95 % CI −0.0004 to 0.00001) with every 1 m increase in elevation. SCR was also highly variable by EAs with a similar mean elevation (Fig. 4a). For one EA in particular, SCR was significantly higher than those of the same altitude range. However, when stratifying the SCR by altitude band, those residing at an elevation between 1550 and 1665 m did show a significantly lower SCR compared to those residing at the lower elevations (Fig. 5a). Despite the overall RDT (Fig. 4b) and PCR (Fig. 4c) prevalence being low, no linear trend (increase of ~0 per increase in 1 m of elevation; RDT, p = 0.99; PCR, p = 0.32) was observed between EAs and elevation.

Next, seroprevalence curves were calculated based on the different reported mosquito control interventions. As would be expected, the group reporting having both received IRS and having slept under a bed net the previous night showed evidence of lower exposure compared to those reporting using a single intervention (Fig. 5b; data shown in Additional file 1). Interestingly, the exposure in groups having reported using a single mosquito control intervention, either bed nets or IRS, showed no difference compared to the group that reported using no malaria control interventions (Fig. 5b).

Factors associated with malaria infection by RDT and PCR, and malaria exposure, defined as MSP seropositivity, were also assessed. The unadjusted analysis for RDT positivity suggested that older age was associated with reduced odds of infection whereas reporting use of IRS and residing in a house with open eaves increased the odds of being RDT positive (Table 4). Next, an adjusted analysis for RDT positivity was conducted (Table 5). Children between 5 and 10 years of age showed the highest odds (AOR 1.81; 95 % CI 1.16–2.81; p = 0.009) of being infected compared to those under 5 years, whilst adults over 20 years were the least likely to be infected (AOR 0.27; 95 % CI 0.12–0.62; p = 0.003). Also, residing in a house with open eaves was associated with five times the odds of RDT infection (AOR 5.05; 95 % CI 1.6–16.0; p = 0.007). Use of mosquito control was also associated with having a patent infection with only those reporting use of IRS only being significant (AOR 2.61; 95 % CI 1.01–6.78; p = 0.48).

**Table 4:** Univariable logistic regression analysis of factors associated with infection and exposure to *Plasmodium falciparum*

	RDT			PCR			MSP		
	OR	95 % CI	P value	OR	95 % CI	P value	OR	95 % CI	P value
Age group in years									
≤5	1	–	–	1	–	–	1	–	–
5.1–10	1.5	0.98–2.4	0.058	1.78	1.2–2.4	0.001	1.48	1.11–1.97	<0.001
10.1–15	0.97	0.57–1.65	0.91	1.37	0.91–2.04	0.125	2.77	2.1–3.68	<0.001
15.1–20	0.63	0.27–1.47	0.28	1.41	0.79–2.51	0.24	4.69	3.51–6.27	<0.001
>20	0.21	0.094–0.5	0.0001	0.75	0.51–1.1	0.141	7.56	5.97–9.58	<0.001
Sex (female)	0.88	0.60–1.29	0.505	0.74	0.58–0.95	0.021	1.41	1.21–1.64	<0.001
Anaemia (<11 g/dl)	2.7	1.75–4.25	0.0001	1.03	0.77–1.3	0.82	0.99	0.81–1.22	0.95
Socio-economic status (SES) score									
1 (low)	1	–	–	1	–	–	1	–	–
2	0.64	0.31–1.31	0.22	0.70	0.44–1.10	0.11	1.04	0.77–1.41	0.78
3	0.65	0.33–1.25	0.19	0.91	0.56–1.5	0.71	0.80	0.63–1.02	0.072
4	0.30	0.11–0.77	0.014	0.63	0.39–1.05	0.075	0.87	0.64–1.19	0.37
5 (high)	0.45	0.21–0.93	0.033	0.84	0.55–1.27	0.407	0.81	0.62–1.07	0.13
Open eaves	6.04	1.8–20.1	0.004	1.2	0.9–1.6	0.19	0.88	0.62–1.2	0.5
Travel	0.18	0.028–1.13	0.066	0.58	0.24–1.40	0.218	1.34	0.99–1.83	0.059
Mosquito control									
None	1	–	–	1	–	–	1	–	–
Net only	0.95	0.45–1.97	0.88	1.19	0.81–1.77	0.363	1.04	0.77–1.41	0.80
IRS only	2.5	1.01–6.15	0.05	2.01	1.13–3.56	0.02	1.13	0.81–1.59	0.46
Both	1.13	0.50–2.57	0.76	1.33	0.87–2.04	0.18	0.79	0.59–1.05	0.106
Elevation (m)									
1430–1494	1	–	–	1	–	–	1	–	–
1495–1510	0.65	0.27–1.57	0.332	0.80	0.48–1.34	0.389	1.02	0.76–1.35	0.905
1511–1529	0.83	0.44–1.58	0.571	1.24	0.72–2.13	0.419	0.95	0.70–1.29	0.749
1530–1685	0.68	0.31–1.46	0.314	1.11	0.64–1.92	0.714	0.68	0.52–0.77	<0.001

**Table 5:** Multivariate logistic regression analysis of factors associated with infection and exposure to *Plasmodium falciparum*

	RDT			PCR			MSP		
	AOR	95 % CI	P value	AOR	95 % CI	P value	AOR	95 % CI	P value
Age group in years									
≤5	1	–	–	1	–	–	1	–	–
5.1–10	1.81	1.17–2.81	0.009	1.73	1.22–2.45	0.003	1.57	1.17–2.11	0.003
10.1–15	1.45	0.83–2.51	0.182	1.48	0.99–2.19	0.052	2.95	2.24–3.89	<0.001
15.1–20	0.86	0.37–2.02	0.726	1.28	0.68–2.42	0.438	5.11	3.85–6.79	<0.001
>20	0.27	0.12–0.62	0.003	0.77	0.52–1.13	0.180	8.18	6.51–10.27	<0.001
Anaemic (<11 g/dl)	2.91	1.88–4.53	<0.001				1.26	1.02–1.56	0.034
Elevation (m)									
1430–1494							1.0	–	–
1495–1510							1.0	0.72–1.38	0.995
1511–1529							0.94	0.65–1.35	0.724
1530–1685							0.63	0.46–0.86	0.005
Socio-economic status (SES) score									
1 (low)	1	–	–	1	–	–	1	–	–
2	0.53	0.20–1.46	0.217	0.57	0.35–0.94	0.030	1.07	0.77–1.50	0.674
3	0.57	0.23–1.42	0.223	0.78	0.44–1.38	0.385	0.80	0.62–1.03	0.081
4	0.30	0.10–0.93	0.038	0.58	0.33–1.02	0.059	0.83	0.59–1.17	0.290
5 (high)	0.64	0.26–1.59	0.326	0.74	0.44–1.22	0.232	0.69	0.52–0.92	0.013
Open eaves	5.05	1.60–16.0	0.007						
Mosquito control									
None	1	–	–	1	–	–			
Net only	1.40	0.49–4.01	0.516	1.54	0.98–2.43	0.060			
IRS only	2.61	1.01–6.78	0.048	2.13	1.21–3.75	0.010			
Both	1.49	0.52–4.29	0.446	1.67	1.00–2.79	0.051			

In the unadjusted analysis using PCR positivity as the response variable, results were similar to those of RDT positivity. There were increased odds of infection in older children and in those residing in households reporting use of IRS only, whereas females were less likely to be infected (Table 4). In the adjusted analysis, PCR infections were greater in children aged 5–10 years compared to children <5 years (AOR 1.73; 95 % CI 1.22–2.45). Wealthy households also exhibited lower odds of PCR infection compared to the poorest households although not all SES categories were statistically significant (Table 5). Finally, there was borderline evidence (p = 0.051) to suggest that reporting use of both IRS and bed nets had greater odds of PCR infection than those using no interventions (AOR 1.67; 95 % CI 1.00–2.79). Those who reported using only IRS had over twice the odds of being PCR positive (AOR 2.13; 95 % CI 1.21–3.75) compared to those with no vector control.

As expected for antibodies to *P. falciparum* MSP-1_19_, age was significantly associated with seropositivity (Table 4). As seen in Fig. 5a, a significant difference in seropositivity was observed only between EAs at the highest altitude compared to the lowest (OR 0.68, 95 % CI 0.52–0.77). Females also had increased odds of being seropositive (OR 1.41, 95 % CI 1.21–1.64). In the adjusted analysis those greater than 20 years had 8.18 times the odds of being seropositive compared to children under five (95 % CI 6.51–10.27; p < 0.001). Also, being anaemic was associated with increased odds of being seropositive (AOR 1.26; 95 % CI 1.02–1.56; p = 0.034). Both elevation and SES were associated with decreased odds of seropositivity (Table 5). Those residing at the highest altitude had 35 % reduction in odds of being seropositive (AOR 0.63; 95 % CI 0.46–0.86; p = 0.005) compared to those living in the lowest elevation band. Similarly, those in the highest SES quintile had an AOR of 0.60 (95 % CI 0.52–0.92; p = 0.013) compared to the poorest quintile.

## Discussion

In many areas, as malaria control interventions are scaled up, transmission is being reduced, resulting in an epidemiological transition with changes in disease burden [1]. Factors associated with these transitions are likely multifaceted and due partly to both curative (increased availability and use of RDT and artemisinin-based combination therapy) and preventative measures (increased coverage of ITNs and IRS) [29–31]. As transmission decreases, surveys such as this one conducted in the western Kenyan highlands are important to understand the current local malaria epidemiology, describe the heterogeneity of transmission and identify areas where transmission persists to allow better targeting of control interventions. In this cross-sectional survey, three different but complimentary tools to assess malaria transmission were assessed: RDTs to determine those *P. falciparum* infections that would be targeted for treatment, PCR to identify current infections including those of low level parasitaemia and serology to describe historic exposure. The results here describe a large range in malaria exposure within this area of nominally low transmission and that the more sensitive diagnostics, using PCR to detect parasite DNA and ELISA to detect antibodies to malaria, provide added value in terms of understanding the epidemiology in this setting and determining risk factors for malaria.

RDTs are useful in a clinical setting and can provide a crude metric for malaria prevalence. However, not unexpectedly, in this study a large proportion of the infections that were detected using the more sensitive PCR metric were missed by RDT. The presence of a large reservoir of sub-patent infections is common in low transmission settings where parasite densities tend to be below the detection threshold of RDTs or microscopy [10]. Therefore, the more sensitive PCR metric provides an accurate estimate of the parasite reservoir in the community, the identification of which is essential when considering malaria elimination [32]. The expense of the equipment and reagents and time taken to conduct current versions of molecular assays limit use as a routine diagnostic test [33], however these tools can identify which sections of the population harbour sub-patent infections and therefore could be targeted for community-level control. For example, in this study the highest levels of sub-patent infection were in those over the age of 15 years.

Seroprevalence to the *P. falciparum* MSP antigen provided insight into malaria exposure levels in the study area. SCR has been associated with the entomological inoculation rate (EIR), which provides a measure of transmission intensity [23]. The wide range of SCR as well as the range of seroprevalence in children <5 years of age suggests that malaria exposure is highly variable in this area. As a measure that integrates exposure over time and reflects cumulative exposure rather than a single current infection, serology can provide a more robust picture of the malaria transmission dynamics in an area. In this setting, in the EAs with no evidence of infections by RDT, serological and molecular tools enabled a more complete understanding of the ongoing transmission and allowed for an examination of risk factors. Serological outcomes can supplement metrics of current infection and be used to identify risk factors where transmission is low [34, 35].

In this population, consistent with other findings, age and wealth indicators were both associated with malaria infection by all metrics [36]. Children between 5 and 10 years of age were more likely to be positive by both RDT and PCR compared to children under 5 years of age, whilst those over 20 years had reduced odds of RDT-confirmed infection. Those of higher SES were less likely to be infected with or show historical exposure to malaria. The presence of open eaves on households was also associated with being RDT positive, as would be expected due to increased house entry of the vector [37, 38]. House screening has been shown to reduce mosquito entry and reduce the odds of malaria infection [39–41].

Interestingly, altitude of residence of survey participants was not associated with either measure of current infection in the adjusted analysis and only with malaria exposure, determined by serology, at the highest altitudes. This lack of association may be due to the low RDT and PCR prevalence in this population or due to the pre-selected narrow altitude range. However, this suggests that the association with altitude and current infection may be more useful at a larger spatial scale [42] or that in this setting, measures of current infection may be less stable on such a short time scale but is consistent with altitude over longer time periods. Also, recent travel was not associated with any malaria metric even though travel is consistently seen as a risk factor for malaria [43]. However, travel, as a risk factor for malaria, will be dependent on who is travelling and their destination in terms of changes in risk. Previous studies have shown those with higher SES are more likely to travel and may have a lower overall risk of malaria infection through increased knowledge and use of protective measures. Also, it has been found that the majority of people in this population travel to Nairobi, the capital city, which has a low or negligible risk of malaria compared to the Kisii and Rachuonyo [44]. Therefore, the lack of association with travel is not surprising.

Regression analyses suggested that those who had received IRS in the past 12 months had an increased risk of being malaria positive at the time of the survey. Although this is at first counter-intuitive, this finding is likely confounded by the fact that areas with higher perceived risk tend to be the ones more like to be targeted and receptive to the use of mosquito control [45]. In 2009 areas considered to be of higher risk in Kenya were targeted for the IRS campaign. Also, that year the IRS campaign was conducted after onset of the rains; at the time of the survey the current campaign was nearing completion. The RDT and PCR results may therefore have not measured any benefit of the campaign that year.

It was observed that those who reported using both ITN and IRS resided in a cluster with a significantly lower SCR than those using just a single intervention. This suggests that combining interventions may be more efficient at reducing exposure and driving transmission to even lower levels, as demonstrated in modelling studies [46–48]. Data from studies in Bioko Island, lowland Kenya and Tanzania indicated combining ITN and IRS resulted in lower prevalence and incidence of malaria than those who received only nets [19, 49–51]. However, other trials and observational studies in Africa indicate little or no added benefit of combining interventions [52–55]. As each intervention is scaled up within Kenya and across the continent, it is increasingly likely that people will be protected by both ITN and IRS [56] and therefore the synergistic effect of the combined intervention coverage should be further explored [54].

One limitation of the serology component of this study was the use of only one antigen (which was all that was available at the time) to assess anti-malarial antibody responses. Increasing the number of antigens would most likely lead to marginal increases in the overall seroprevalence estimates due to heterogeneous immunity in the population. However, whilst this means that the serological outcomes presented here can be assumed to be underestimates, responses to non-variant antigens such as MSP-1_19_ with a comparatively long antibody half-life [57] should be considered reasonably robust [58]. Furthermore, it is acknowledged that the small sample sizes within each cluster affects the precision of the estimated cluster-level SCR and are highlighted in the width of the reported confidence intervals. Calculations of SCR would be more robust with standardized age ranges per cluster but as the household survey was designed and powered to estimate parasite rate, and given that the sampling framework included all ages and the SRR was fixed, the bias is minimized [24].

In this study, EAs were sampled from two districts: Kisii is classified as having low and epidemic-prone malaria transmission whereas Rachuonyo has low and stable transmission. However, analysis of malaria burden determined by all metrics at the EA level showed no clear differences, with those with the highest burden being equally in both districts. The cluster with the highest SCR was in fact located in Kisii, the district with lower overall reported levels of malaria burden (although the low sample sizes used to estimate SCR on the EA level are acknowledged). This suggests that allocating resources at the district level may not be ideal; pockets of high burden within the overall low transmission area would be missed and may seed transmission to neighbouring areas [59]. Although it is logistically more difficult to deploy malaria control interventions at a more refined spatial scale, such efforts may prove to be more efficient as interventions could be more precisely targeted and prove to be more cost-effective than blanket treatment. Therefore, strong health systems and surveillance based on sensitive diagnostic tools, as well as the capacity to analyse the data collected, will be necessary for informed decisions to be made at a time scale that is relevant for malaria control.

## Conclusion

Ultimately, in the highlands in western Kenya, malaria transmission is generally low, but highly heterogeneous and difficult to measure accurately using routine diagnostic tools. This study found a large range in malaria burden at the EA level and a large population of sub-patent infections. This suggests that for malaria surveillance and monitoring, more sensitive metrics such as molecular or serological methods may complement current diagnostic tools in order to accurately assess malaria burden in the population, determine reservoirs of infection, identify risk factors and, therefore, better target appropriate interventions.

## Additional file

10.1186/s12936-015-0944-4 Seroconversion rates (SCR) and corresponding 95 % confidence interval (CI) stratified by elevation and mosquito control categories. The table shows the seroconversion rates by elevation and mosquito control category, demonstrating lower exposure to malaria at altitudes above 1530 m and in households with both IRS and ITNs in their households.

## References

[1] Cotter C, Sturrock HJW, Hsiang MS, Liu J, Phillips AA, Hwang J (2013). The changing epidemiology of malaria elimination: new strategies for new challenges. Lancet.

[2] Breman JG, Brandling-Bennett AD (2011). The challenge of malaria eradication in the twenty-first century: research linked to operations is the key. Vaccine.

[3] Griffin JT, Hollingsworth TD, Okell LC, Churcher TS, White M, Hinsley W (2010). Reducing *Plasmodium falciparum* malaria transmission in Africa: a model-based evaluation of intervention strategies. PLoS Med.

[4] Tusting LS, Bousema T, Smith DL, Drakeley C (2014). Measuring changes in *Plasmodium falciparum* transmission: precision, accuracy and costs of metrics. Adv Parasitol.

[5] Okell L, Bousema T, Griffin J, Ouedraogo A, Ghani A, Drakeley C (2012). Factors determining the occurrence of submicroscopic malaria infections and their relevance for control. Nat Commun.

[6] Okell L, Ghani A, Lyons E, Drakeley C (2009). Submicroscopic infection in *Plasmodium falciparum*-endemic populations: a systematic review and meta-analysis. J Infect Dis.

[7] Laban N, Kobayashi T, Hamapumbu H, Sullivan D, Mharakurwa S, Thuma P (2015). Comparison of a PfHRP2-based rapid diagnostic test and PCR for malaria in a low prevalence setting in rural southern Zambia: implications for elimination. Malar J.

[8] Manjurano A, Okell L, Lukindo T, Reyburn H, Olomi R, Roper C (2011). Association of sub-microscopic malaria parasite carriage with transmission intensity in north-eastern Tanzania. Malar J.

[9] Greenhouse B, Ho B, Hubbard A, Njama-Meya D, Narum DL, Lanar DE (2011). Antibodies to *Plasmodium falciparum* antigens predict a higher risk of malaria but protection from symptoms once parasitemic. J Infect Dis.

[10] Lindblade KA, Steinhardt L, Samuels A, Kachur SP, Slutsker L (2013). The silent threat: asymptomatic parasitemia and malaria transmission. Expert Rev Anti Infect Ther.

[11] Reyburn H, Mbatia R, Drakeley C, Bruce J, Carneiro I, Olomi R (2005). Association of transmission intensity and age with clinical manifestations and case fatality of severe *Plasmodium falciparum* malaria. JAMA.

[12] Lo E, Zhou G, Oo W, Afrane Y, Githeko A, Yan G (2015). Low parasitemia in submicroscopic infections significantly impacts malaria diagnostic sensitivity in the highlands of Western Kenya. PLoS One.

[13] Bousema T, Youssef RM, Cook J, Cox J, Alegana VA, Amran J (2010). Serologic markers for detecting malaria in areas of low endemicity, Somalia, 2008. Emerg Infect Dis.

[14] Drakeley CJ, Corran PH, Coleman PG, Tongren JE, McDonald SL, Carneiro I (2005). Estimating medium- and long-term trends in malaria transmission by using serological markers of malaria exposure. Proc Natl Acad Sci USA.

[15] Lindblade KA, Eisele TP, Gimnig JE, Alaii JA, Odhiambo F, ter Kuile FO (2004). Sustainability of reductions in malaria transmission and infant mortality in western Kenya with use of insecticide-treated bednets: 4–6 years of follow-up. JAMA.

[16] Ototo EN, Githeko AK, Wanjala CL, Scott TW (2011). Surveillance of vector populations and malaria transmission during the 2009/10 El Nino event in the western Kenya highlands: opportunities for early detection of malaria hyper-transmission. Parasit Vectors.

[17] Wanjala CL, Waitumbi J, Zhou G, Githeko AK (2011). Identification of malaria transmission and epidemic hotspots in the western Kenya highlands: its application to malaria epidemic prediction. Parasit Vectors.

[18] Githeko AK, Ototo EN, Guiyun Y (2012). Progress towards understanding the ecology and epidemiology of malaria in the western Kenya highlands: opportunities and challenges for control under climate change risk. Acta Trop.

[19] Hamel M, Otieno P, Bayoh N, Kariuki S, Were V, Marwanga D (2011). The combination of indoor residual spraying and insecticide-treated nets provides added protection against malaria compared with insecticide-treated nets alone. Am J Trop Med Hyg.

[20] Odhiambo FO, Laserson KF, Sewe M, Hamel MJ, Feikin DR, Adazu K (2012). Profile: the KEMRI/CDC health and demographic surveillance system—Western Kenya. Int J Epidemiol.

[21] Stuckey E, Stevenson J, Cooke M, Owaga C, Marube E, Oando G (2012). Simulation of malaria epidemiology and control in the highlands of western Kenya. Malar J.

[22] MoPHS. National guidelines for HIV testing and counselling in Kenya—2010. In: Programme NAaSC. Nairobi, Kenya: Ministry of Public Health and Sanitation. 2010.

[23] Corran P, Coleman P, Riley E, Drakeley C (2007). Serology: a robust indicator of malaria transmission intensity?. Trends Parasitol.

[24] Sepulveda N, Drakeley C (2015). Sample size determination for estimating antibody seroconversion rate under stable malaria transmission intensity. Malar J.

[25] Baidjoe A, Stone W, Ploemen I, Shagari S, Grignard L, Osoti V (2013). Combined DNA extraction and antibody elution from filter papers for the assessment of malaria transmission intensity in epidemiological studies. Malar J.

[26] Snounou G, Viriyakosol S, Zhu XP, Jarra W, Pinheiro L, do Rosario VE (1993). High sensitivity of detection of human malaria parasites by the use of nested polymerase chain reaction. Mol Biochem Parasitol..

[27] Steenkeste N, Incardona S, Chy S, Duval L, Ekala MT, Lim P (2009). Towards high-throughput molecular detection of Plasmodium: new approaches and molecular markers. Malar J.

[28] Stevenson JC, Stresman GH, Gitonga CW, Gillig J, Owaga C, Marube E (2013). Reliability of school surveys in estimating geographic variation in malaria transmission in the western Kenyan highlands. PLoS One.

[29] Karema C, Aregawi MW, Rukundo A, Kabayiza A, Mulindahabi M, Fall IS (2012). Trends in malaria cases, hospital admissions and deaths following scale-up of anti-malarial interventions, 2000–2010, Rwanda. Malar J.

[30] Mharakurwa S, Mutambu SL, Mberikunashe J, Thuma PE, Moss WJ, Mason PR (2013). Changes in the burden of malaria following scale up of malaria control interventions in Mutasa District, Zimbabwe. Malar J.

[31] O’Meara WP, Bejon P, Mwangi TW, Okiro EA, Preshu N, Snow RW (2008). Effect of a fall in malaria transmission on morbidity and mortality in Kilifi, Kenya. Lancet.

[32] Moonen B, Cohen JM, Snow RW, Slutsker L, Drakeley C, Smith DL (2010). Operational strategies to achieve and maintain malaria elimination. Lancet.

[33] McMorrow ML, Aidoo M, Kachur SP (2011). Malaria rapid diagnostic tests in elimination settings—can they find the last parasite?. Clin Microbiol Infect.

[34] Cook J, Reid H, Iavro J, Kuwahata M, Taleo G, Clements A (2010). Using serological measures to monitor changes in malaria transmission in Vanuatu. Malar J.

[35] Cook J, Kleinschmidt I, Schwabe C, Nseng G, Bousema T, Corran PH (2011). Serological markers suggest heterogeneity of effectiveness of malaria control interventions on Bioko Island, Equatorial Guinea. PLoS One.

[36] Tusting LS, Willey B, Lucas H, Thompson J, Kafy HT, Smith R (2013). Socioeconomic development as an intervention against malaria: a systematic review and meta-analysis. Lancet.

[37] Kirby MJ, Green C, Milligan PM, Sismanidis C, Jasseh M, Conway DJ (2008). Risk factors for house-entry by malaria vectors in a rural town and satellite villages in The Gambia. Malar J.

[38] Njie M, Dilger E, Lindsay SW, Kirby MJ (2009). Importance of eaves to house entry by anopheline, but not culicine, mosquitoes. J Med Entomol.

[39] Kirby MJ, Ameh D, Bottomley C, Green C, Jawara M, Milligan PJ (2009). Effect of two different house screening interventions on exposure to malaria vectors and on anaemia in children in The Gambia: a randomised controlled trial. Lancet.

[40] Ghebreyesus TA, Haile M, Witten KH, Getachew A, Yohannes M, Lindsay SW (2000). Household risk factors for malaria among children in the Ethiopian highlands. Trans R Soc Trop Med Hyg.

[41] Wanzirah H, Tusting LS, Arinaitwe E, Katureebe A, Maxwell K, Rek J (2015). Mind the gap: house structure and the risk of malaria in Uganda. PLoS One.

[42] Githeko AK, Ayisi JM, Odada PK, Atieli FK, Ndenga BA, Githure JI (2006). Topography and malaria transmission heterogeneity in western Kenya highlands: prospects for focal vector control. Malar J.

[43] Wesolowski A, Eagle N, Tatem AJ, Smith DL, Noor AM, Snow RW (2012). Quantifying the impact of human mobility on malaria. Science.

[44] Wesolowski A, Stresman G, Eagle N, Stevenson J, Owaga C (2014). Quantifying travel behavior for infectious disease research: a comparison of data from surveys and mobile phones. Sci Rep.

[45] Kolaczinski K, Kolaczinski J, Kilian A, Meek S (2007). Extension of indoor residual spraying for malaria control into high transmission settings in Africa. Trans R Soc Hyg Trop Med.

[46] Yakob L, Dunning R, Yan G (2010). Indoor residual spray and insecticide treated bednets for malaria control: theoretical synergisms and antagonisms. J R Soc Interface.

[47] Chitnis N, Schapira A, Smith T, Steketee R (2010). Comparing the effectiveness of malaria vector-control interventions through a mathematical model. Am J Trop Med Hyg.

[48] White MT, Griffin JT, Churcher TS, Ferguson NM, Basanez MG, Ghani AC (2011). Modelling the impact of vector control interventions on *Anopheles gambiae* population dynamics. Parasit Vectors.

[49] Kleinschmidt I, Schwabe C, Shiva M, Segura J, Sima V, Mabunda S (2009). Combining indoor residual spraying and insecticide-treated net interventions. Am J Trop Med Hyg.

[50] Kleinschmidt I, Torrez M, Schwabe C, Benavente L, Seocharan I, Jituboh D (2007). Factors influencing the effectiveness of malaria control in Bioko Island, Equatorial Guinea. Am J Trop Med Hyg.

[51] West PA, Protopopoff N, Wright A, Kivaju Z, Tigererwa R, Mosha FW (2014). Indoor residual spraying in combination with insecticide-treated nets compared to insecticide-treated nets alone for protection against malaria: a cluster randomised trial in Tanzania. PLoS Med.

[52] Corbel V, Akogbeto M, Damien G, Djenontin A, Chandre F, Rogier C (2012). Combination of malaria vector control interventions in pyrethroid resistance area in Benin: a cluster randomized controlled trial. Lancet Infect Dis.

[53] Protopopoff N, Van Bortel W, Marcotty T, Van Herp M, Maes P, Baza D (2008). Spatial targeted vector control is able to reduce malaria prevalence in the highlands of Burundi. Am J Trop Med Hyg.

[54] Okumu F, Moore S (2011). Combining indoor residual spraying and insecticide-treated nets for malaria control in Africa: a review of possible outcomes and an outline of suggestions for the future. Malar J.

[55] Fullman N, Burstein R, Lim S, Medlin C, Gakidou E (2013). Nets, spray or both? The effectiveness of insecticide-treated nets and indoor residual spraying in reducing malaria morbidity and child mortality in sub-Saharan Africa. Malar J.

[56] Noor AM, Amin AA, Akhwale WS, Snow RW (2007). Increasing coverage and decreasing inequity in insecticide-treated bed net use among rural Kenyan children. PLoS Med.

[57] Helb DA, Tetteh KKA, Felgner PL, Skinner J, Hubbard A, Arinaitwe E (2015). Novel serologic biomarkers provide accurate estimates of recent *Plasmodium falciparum* exposure for individuals and communities. Proc Natl Acad Sci USA.

[58] Bejon P, Turner L, Lavstsen T, Cham G, Olotu A, Drakeley CJ (2011). Serological evidence of discrete spatial clusters of *Plasmodium falciparum* parasites. PLoS One.

[59] Bousema T, Griffin JT, Sauerwein RW, Smith DL, Churcher TS, Takken W (2012). Hitting hotspots: spatial targeting of malaria for control and elimination. PLoS Med.

