# Supplementary material for: Use of different transmission metrics to describe malaria epidemiology in the highlands of western Kenya
Source: Malar J. 2015 Oct 26;14:418. doi: 10.1186/s12936-015-0944-4 (PMC4624380; doi:10.1186/s12936-015-0944-4)
Supplement: Supplementary file 1 — 10.1186/s12936-015-0944-4 Seroconversion rates (SCR) and corresponding 95 % confidence interval (CI) stratified by elevation and mosquito control categories. The table shows the seroconversion rates by elevation and mosquito control category, demonstrating lower exposure to malaria at altitudes above 1530 m and in households with both IRS and ITNs in their households. [file 12936_2015_944_MOESM1_ESM.docx]

**Additional file 1 Seroconversion rates (SCR) and corresponding 95% confidence interval (CI) stratified by elevation and mosquito control categories**

Bolded values highlight categories that are significantly different than other groups.

|  | SCR | 95% CI |
| --- | --- | --- |
| Elevation | | |
| Combined | 0.038 | 0.035-0.040 |
| 1,430-1,494 m | 0.041 | 0.037-0.046 |
| 1,495-1,510 m | 0.042 | 0.037-0.049 |
| 1,511-1,529 m | 0.040 | 0.035-0.045 |
| **1,530-1,685 m** | **0.029** | **0.025-0.033** |
| Mosquito control | | |
| Net only | 0.042 | 0.038-0.047 |
| IRS only | 0.045 | 0.037-0.055 |
| **Both** | **0.031** | **0.028-0.035** |
| None | 0.041 | 0.036-0.046 |
